# Supplementary material for: Multi-centre analysis of networks and genes modulated by hypothalamic stimulation in patients with aggressive behaviours
Source: eLife. 2023 May 22;12:e84566. doi: 10.7554/eLife.84566 (PMC10259501; doi:10.7554/eLife.84566)
Supplement: Supplementary file 1. [file elife-84566-supp1.pdf]

Supplementary material accompanying:

Networks and genes modulated by posterior hypothalamic stimulation in patients with aggressive behaviours: Analysis of probabilistic mapping, normative connectomics, and atlas-derived transcriptomics of the largest international multi-centre dataset

By

Flavia Venetucci Gouveia, Jürgen Germann, Gavin JB Elias, Alexandre Boutet, Aaron Loh, Adriana Lucia Lopez Rios, Cristina V Torres Diaz, William Omar Contreras Lopez, Raquel CR Martinez, Erich T Fonoff, Juan C Benedetti-Isaac, Peter Giacobbe, Pablo M Arango Pava, Han Yan, George M Ibrahim, Nir Lipsman, Andres M Lozano, Clement Hamani

Corresponding Authors:

Dr. Flavia Venetucci Gouveia. Neuroscience and Mental Health, Hospital for Sick Children. 686, Bay Street, Toronto, ON, M5G 0A4, Canada. flavia.venetuccigouveia@sickkids.ca

Dr. Clement Hamani. Sunnybrook Research Institute. 2075 Bayview Ave, S126. Toronto, ON, M4N3M5, Canada. clement.hamani@sunnybrook.ca

SUPPLEMENTARY FIGURE 1

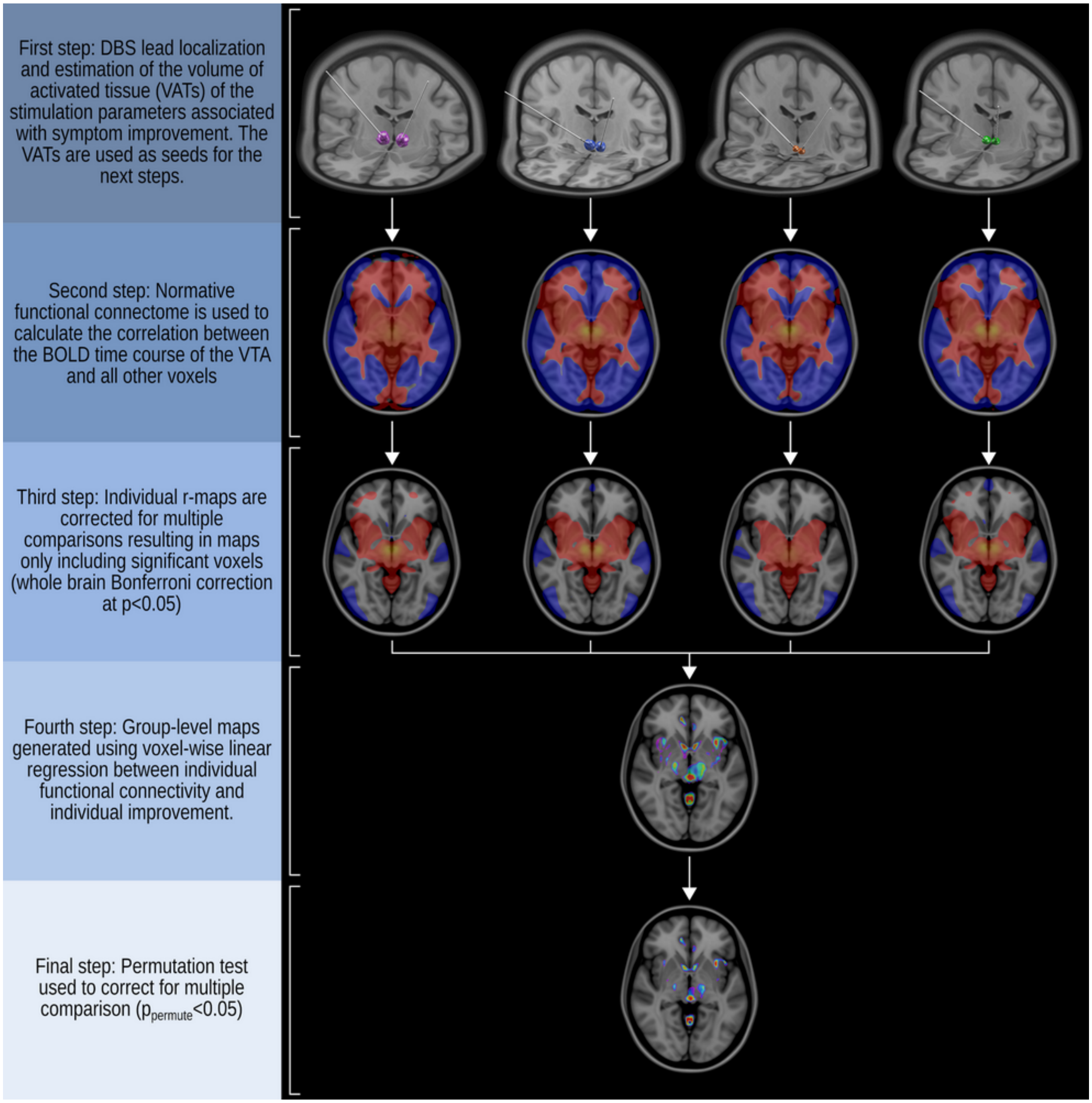

Supplementary Figure 1. Method of generating functional connectivity maps. This process involves the localization of the electrodes in each patient's brain and the estimation of the volume of activated tissue (VAT) based on the stimulation parameters associated with symptom improvement. The VATs are then used as seeds for the generation of an individual r-map by correlating the BOLD time course of the VATs seed with the BOLD time course of all other voxels using the normative data of 1000 subjects (Brain Genomics Superstruct Project dataset, http://neuroinformatics.harvard.edu/gsp). Individual r-maps are corrected for multiple comparisons to exclude voxels with potentially spurious correlations, resulting in an individual r-map that only included voxels surviving Bonferroni correction at the level of $p<0.05$. Finally, to create group-level maps, a voxel-wise linear regression analysis is performed to investigate the relationship between the functional connectivity of the VATs and the individual clinical outcome, followed by permutation correction resulting in a significant group-level functional connectivity map ($p_{permute}<0.05$). The MNI152 brain was used for axial and 3-dimensional brain images (https://www.bic.mni.mcgill.ca/ServicesAtlases/ICBM152NLin2009).

SUPPLEMENTARY FIGURE 2

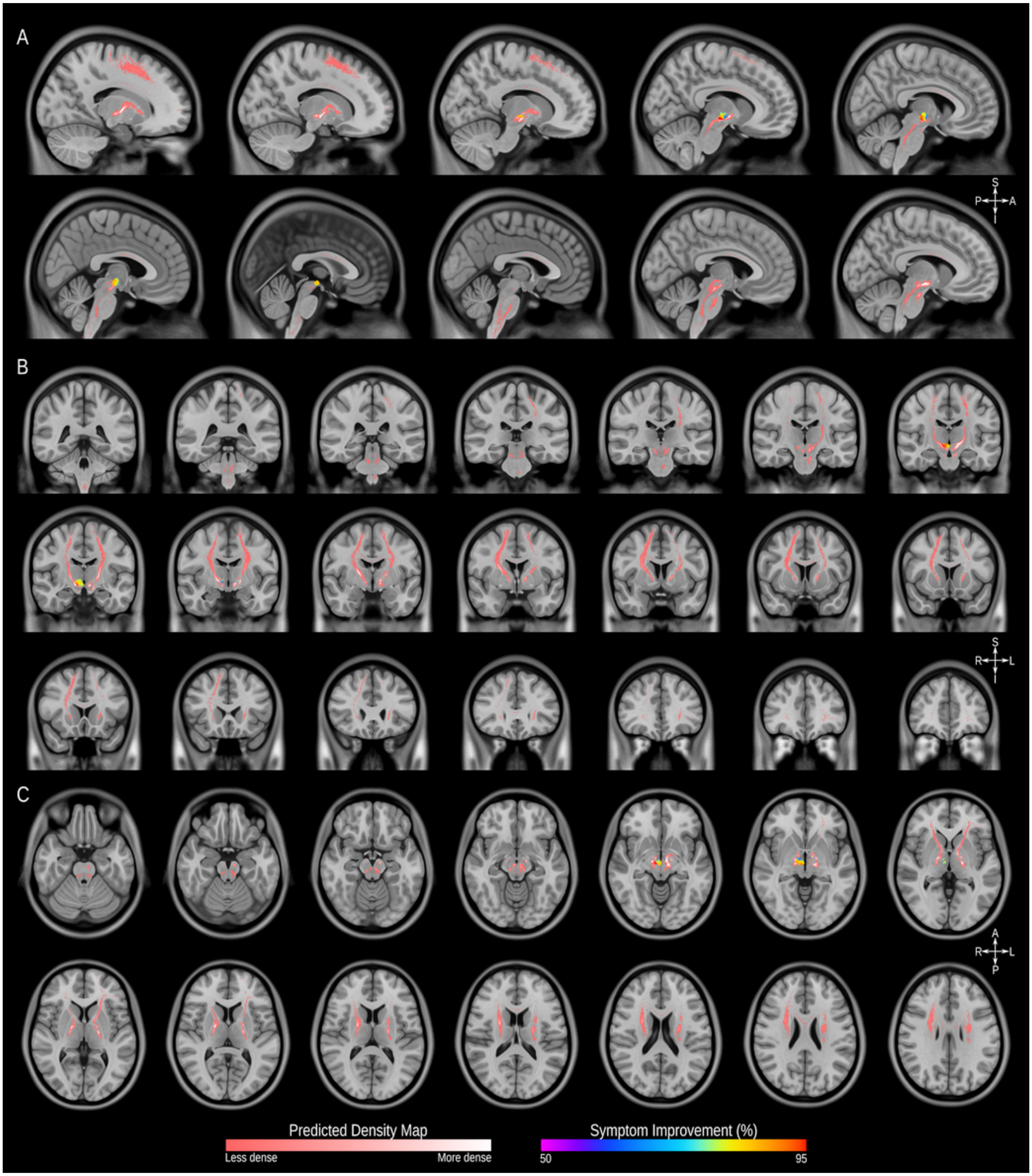

Supplementary Figure 2. Structural Connectivity Mapping. Magnetic Resonance Imaging (MRI) in the sagittal (A), coronal (B) and axial (C) planes showing the fibre density of streamlines (pink scale) streamlines connected to the volumes of activated tissue (VATs) associated with significantly greater symptom improvement and voxels associated with at least 50% improvement in symptoms (heat map), illustrated in the MNI152 brain (https://www.bic.mni.mcgill.ca/ServicesAtlases/ICBM152NLin2009).

SUPPLEMENTARY FIGURE 3

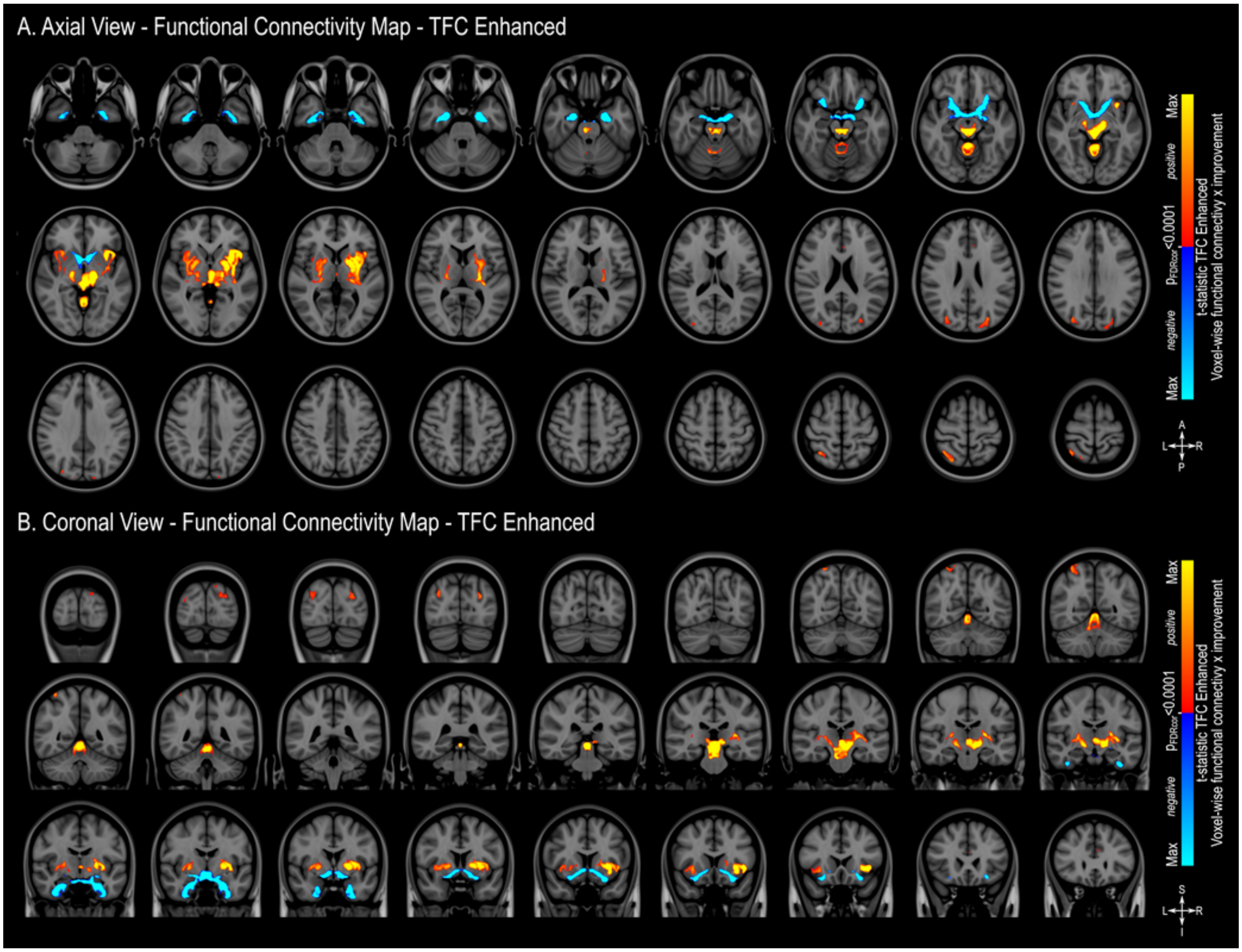

Supplementary Figure 3. Threshold-free Cluster Enhancement Functional Connectivity Mapping. Magnetic Resonance Imaging (MRI) in the axial plane (A) and coronal plane (B) showing areas found to have a positive correlation between clinical outcome and functional connectivity (warm colours) or a negative correlation between outcome and functional connectivity (cold colours) FDR corrected at q<0.0001. The results are illustrated in the MNI152 brain (https://www.bic.mni.mcgill.ca/ServicesAtlases/ICBM152NLin2009).

SUPPLEMENTARY FIGURE 4

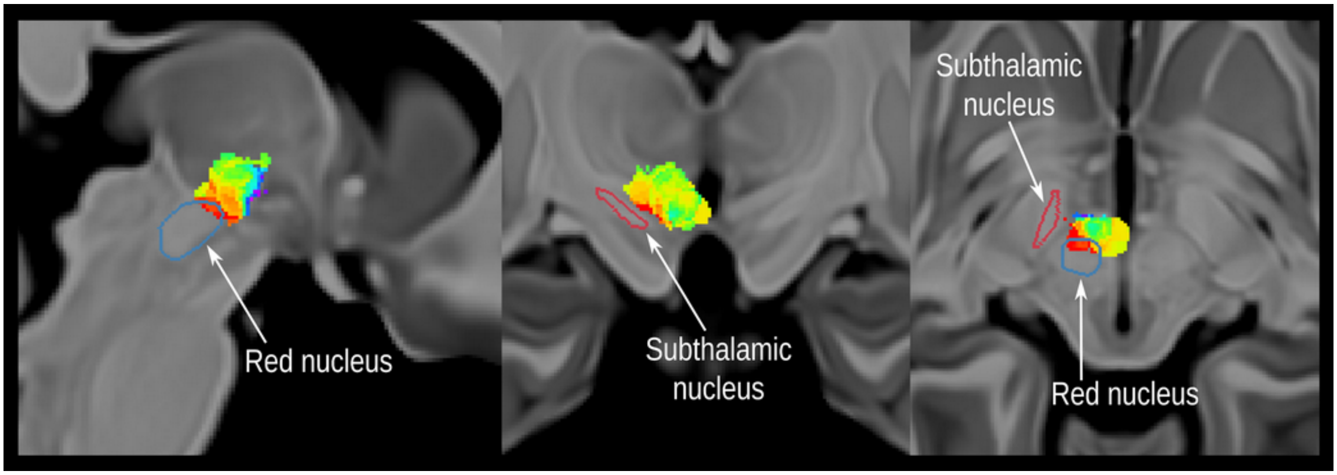

Supplementary Figure 4. Localization of the probabilistic sweet spot mapping associated with at least 50% improvement in symptoms, in the posterior-inferior-lateral region of the posterior hypothalamic area, and in relation to the red nucleus and the subthalamic nucleus. Note that a small portion of the map overlaps with the most superior part of the red nucleus, and no overlap with the subthalamic nucleus is observed. The labels for the Subthalamic nucleus and red nucleus are derived from a previously published high-resolution MRI atlas of the human hypothalamic region (https://zenodo.org/record/3903588#.YHiE7pNKiF0), and illustrated in the MNI152 brain (https://www.bic.mni.mcgill.ca/ServicesAtlases/ICBM152NLin2009).

SUPPLEMENTARY FIGURE 5

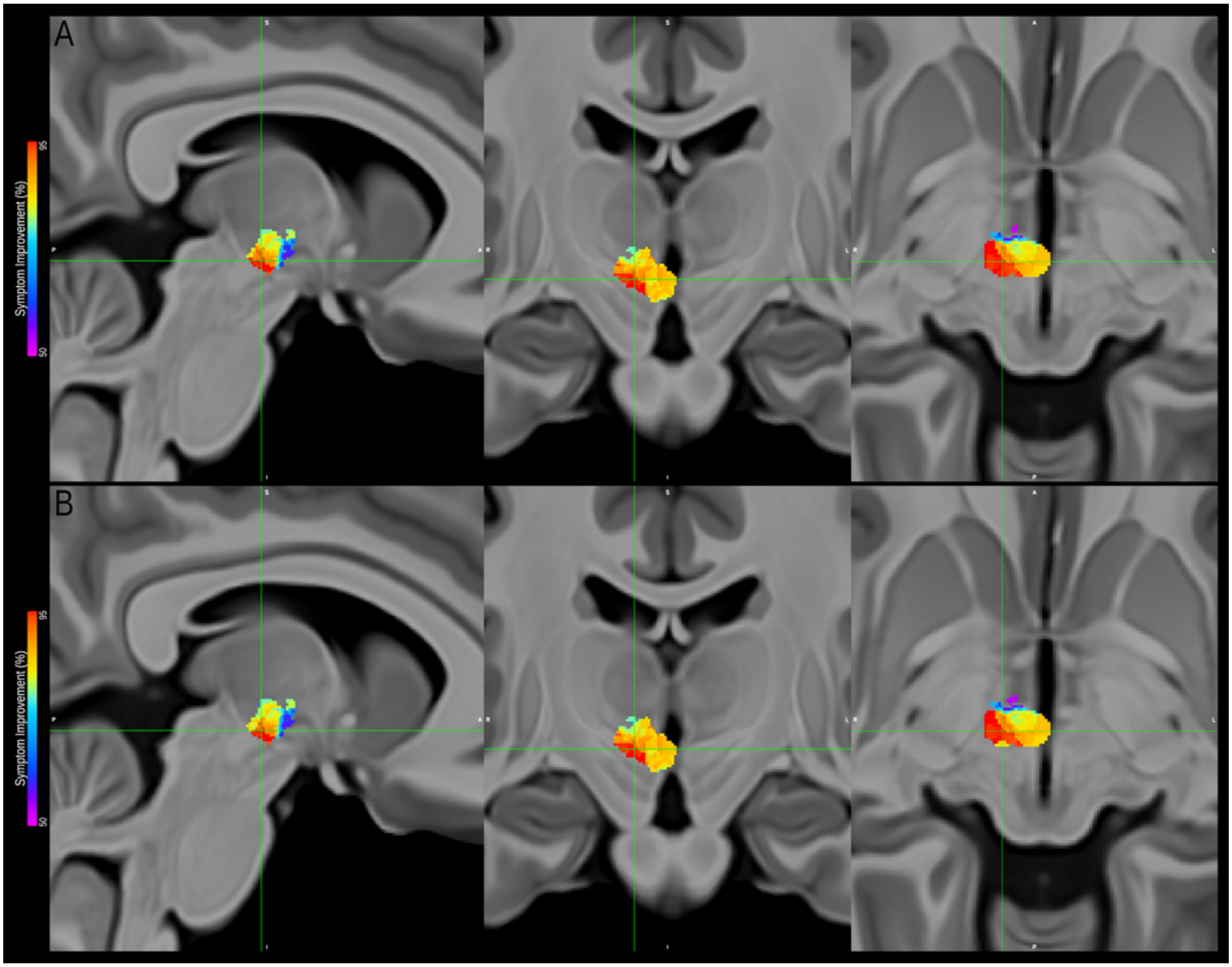

Supplementary Figure 5. Comparison between two probabilistic sweet-spot maps performed considering amplitude (top panel, original analysis) and amplitude plus pulse width (bottom panel, additional analysis). Note the striking similarity between maps, with the location and values of the peak corresponding to the most efficacious area for maximal symptom alleviation remaining unaltered, and only a few voxels on the periphery of the map changing in value by a couple of percentage points.

Supplementary Matrix 1. Matrix of the correlations between estimated symptom improvement (i.e. linear model of age and functional connectivity of the two areas) and the measured improvement.

	L Amg	R Amg	R SPL	L dACC	R rACC	L Claus	L aInsu	L Put	R BNST	L BNST	PAG	Verm	L nAcc	R nAcc	L SZS	R SZS	R Hyp	L Hyp	mRaphe	R OFC	L OFC	R Fusi	L Fusi
L Amg	NA	0.675	0.693	0.684	0.676	0.693	0.675	0.683	0.676	0.676	0.753	0.731	0.676	0.675	0.708	0.728	0.673	0.682	0.725	0.676	0.675	0.693	0.672
R Amg	0.675	NA	0.660	0.678	0.646	0.674	0.665	0.677	0.646	0.646	0.739	0.718	0.646	0.646	0.679	0.705	0.646	0.653	0.707	0.646	0.646	0.677	0.657
R SPL	0.693	0.660	NA	0.703	0.653	0.694	0.680	0.680	0.653	0.653	0.738	0.717	0.653	0.660	0.701	0.709	0.657	0.652	0.708	0.653	0.660	0.682	0.663
L dACC	0.684	0.678	0.703	NA	0.678	0.675	0.654	0.660	0.678	0.678	0.754	0.731	0.678	0.679	0.700	0.705	0.665	0.659	0.712	0.678	0.679	0.690	0.661
R rACC	0.676	0.646	0.653	0.678	NA	0.680	0.663	0.676	0.638	0.638	0.761	0.743	0.638	0.646	0.698	0.708	0.642	0.638	0.733	0.638	0.646	0.669	0.646
L Claus	0.693	0.674	0.694	0.675	0.680	NA	0.672	0.676	0.680	0.680	0.742	0.717	0.680	0.674	0.690	0.706	0.672	0.672	0.703	0.680	0.674	0.704	0.689
L aInsu	0.675	0.665	0.680	0.654	0.663	0.672	NA	0.664	0.663	0.663	0.732	0.713	0.663	0.665	0.686	0.693	0.652	0.646	0.697	0.663	0.665	0.679	0.649
L Put	0.683	0.677	0.680	0.660	0.676	0.676	0.664	NA	0.676	0.676	0.732	0.712	0.676	0.677	0.692	0.696	0.665	0.659	0.698	0.676	0.677	0.690	0.662
R BNST	0.676	0.646	0.653	0.678	0.638	0.680	0.663	0.676	NA	0.638	0.761	0.743	0.638	0.646	0.698	0.708	0.642	0.638	0.733	0.638	0.646	0.669	0.646
L BNST	0.676	0.646	0.653	0.678	0.638	0.680	0.663	0.676	0.638	NA	0.761	0.743	0.638	0.646	0.698	0.708	0.642	0.638	0.733	0.638	0.646	0.669	0.646
PAG	0.753	0.739	0.738	0.754	0.761	0.742	0.732	0.732	0.761	0.761	NA	0.725	0.761	0.740	0.725	0.740	0.726	0.734	0.725	0.761	0.740	0.751	0.729
Verm	0.731	0.718	0.717	0.731	0.743	0.717	0.713	0.712	0.743	0.743	0.725	NA	0.743	0.719	0.704	0.716	0.704	0.711	0.707	0.743	0.719	0.732	0.722
L nAcc	0.676	0.646	0.653	0.678	0.638	0.680	0.663	0.676	0.638	0.638	0.761	0.743	NA	0.646	0.698	0.708	0.642	0.638	0.733	0.638	0.646	0.669	0.646
R nAcc	0.675	0.646	0.660	0.679	0.646	0.674	0.665	0.677	0.646	0.646	0.740	0.719	0.646	NA	0.680	0.706	0.646	0.654	0.709	0.646	0.646	0.677	0.657
L SZS	0.708	0.679	0.701	0.700	0.698	0.690	0.686	0.692	0.698	0.698	0.725	0.704	0.698	0.680	NA	0.687	0.673	0.674	0.694	0.698	0.680	0.708	0.687
R SZS	0.728	0.705	0.709	0.705	0.708	0.706	0.693	0.696	0.708	0.708	0.740	0.716	0.708	0.706	0.687	NA	0.692	0.687	0.708	0.708	0.706	0.721	0.693
R Hyp	0.673	0.646	0.657	0.665	0.642	0.672	0.652	0.665	0.642	0.642	0.726	0.704	0.642	0.646	0.673	0.692	NA	0.632	0.693	0.642	0.646	0.670	0.639
L Hyp	0.682	0.653	0.652	0.659	0.638	0.672	0.646	0.659	0.638	0.638	0.734	0.711	0.638	0.654	0.674	0.687	0.632	NA	0.702	0.638	0.654	0.669	0.633
mRaphe	0.725	0.707	0.708	0.712	0.733	0.703	0.697	0.698	0.733	0.733	0.725	0.707	0.733	0.709	0.694	0.708	0.693	0.702	NA	0.733	0.709	0.725	0.717
R OFC	0.676	0.646	0.653	0.678	0.638	0.680	0.663	0.676	0.638	0.638	0.761	0.743	0.638	0.646	0.698	0.708	0.642	0.638	0.733	NA	0.646	0.669	0.646
L OFC	0.675	0.646	0.660	0.679	0.646	0.674	0.665	0.677	0.646	0.646	0.740	0.719	0.646	0.646	0.680	0.706	0.646	0.654	0.709	0.646	NA	0.677	0.657
R Fusi	0.693	0.677	0.682	0.690	0.669	0.704	0.679	0.690	0.669	0.669	0.751	0.732	0.669	0.677	0.708	0.721	0.670	0.669	0.725	0.669	0.677	NA	0.668
L Fusi	0.672	0.657	0.663	0.661	0.646	0.689	0.649	0.662	0.646	0.646	0.729	0.722	0.646	0.657	0.687	0.693	0.639	0.633	0.717	0.646	0.657	0.668	NA

Abbrevitaions: L: left; R: right; Amg: Amygdala; aIns: Anterior Insula; BNST: Bed Nucleus of the Stria Terminallis; Claus: Claustrum; dACC: Dorsal Anterior Cingulate Cortex; Fusi: Fusiform Gyrus; Hyp: Hypothalamus; mRaphe: medial Raphe nucleus; nAcc: Nucleus Accumbens; OFC: Orbitofrontal Cortex; PAG: Periaqueductal Grey matter; Put: Putamen; rACC: Rostral Anterior Cingulate Cotex; SPL: Superior Parietal Lobule; SZS: Substantia Nigra, Zona Incerta, Subthalamic Nucleus; Verm: Vermis
